# Barriers to and Facilitators of the Use of Mobile Health Apps From a Security Perspective: Mixed-Methods Study

**DOI:** 10.2196/11223

**Published:** 2019-04-16

**Authors:** Leming Zhou, Jie Bao, Valerie Watzlaf, Bambang Parmanto

**Affiliations:** 1 Department of Health Information Management University of Pittsburgh Pittsburgh, PA United States

**Keywords:** confidentiality, privacy, mobile apps, questionnaire

## Abstract

**Background:**

A large number of mobile health (mHealth) apps have been created to help users to manage their health or receive health care services. Many of these mHealth apps have proven to be helpful for maintaining or improving their users’ health. However, many people still choose not to use mHealth apps or only use them for a short period. One of the reasons behind this lack of use is the concern for their health information security and privacy.

**Objective:**

The goal of this study was to determine the relationship between users’ characteristics and their security and privacy concerns and to identify desired security features in mHealth apps, which could reduce these concerns.

**Methods:**

A questionnaire was designed and validated by the research team. This questionnaire was then used to determine mobile app users’ security and privacy concerns regarding personal health data in mHealth apps as well as the security features most users’ desire. A semistructured interview was used to identify barriers to and facilitators of adopting mHealth apps.

**Results:**

In total, 117 randomly selected study participants from a large pool took part in this study and provided responses to the validated questionnaire and the semistructured interview questions. The results indicate that most study participants did have concerns about their privacy when using mHealth apps. They also expressed their preferences regarding several security features in mHealth apps, such as regular password updates, remote wipe, user consent, and access control. An association between their demographic characteristics and their concerns and preferences in security and privacy was identified; however, in most cases, the differences among the different demographic groups were not statistically significant, except for a few very specific aspects. These study participants also indicated that the cost of apps and lack of security features in mHealth apps were barriers for adoption, whereas having free apps, strong but easy-to-use security features, and clear user protection privacy policies might encourage them to use mHealth apps in their health management.

**Conclusions:**

This questionnaire and interview study verified the security and privacy concerns of mHealth app users, identified the desired security and privacy features, and determined specific barriers to and facilitators of users adopting mHealth apps. The results can be used to guide mHealth app developers to create apps that would be welcomed by users.

## Introduction

### Background

In recent years, both ownership of smartphones and the number of available mobile health (mHealth) apps have increased dramatically. According to one study performed by the Pew Research Center in 2018, 95% of Americans owned a mobile phone, and in 2018, 77% of them were smartphones [1] as opposed to 2011, when the share of smartphones among American adults was just 35%. In the same period, many mHealth apps were created and published on app stores. Specifically, by October 2017, roughly 325,000 mHealth apps were available on major app stores [2].

For patients (in a general sense, ie, people who want to maintain or improve their health), mHealth apps can be used to perform tasks such as wellness management, encouraging and monitoring behavior change, health data collection, disease management, self-diagnosis, medication reminders, and rehabilitation schedule management [3,4]. A number of research studies have been performed on mHealth apps and their results indicate that well-designed mHealth apps can empower patients, improve medication adherence, and reduce the cost of health care [5-8].

However, the adoption of mHealth apps in personal health care is still limited. The growth rate of mHealth app downloads dropped dramatically from more than 35% in 2015 to roughly 7% in 2016 [2]. Moreover, it has been shown that after smartphone users download mHealth apps, close to half of them stop using mHealth apps for various reasons such as hidden costs, high data entry burden, loss of interest, and security and privacy concerns [9].

In the context of patient health data, security and privacy are always linked since any unauthorized access to patient health data (security breach) is a violation of patient privacy. Here, security is the state of being protected against the unauthorized use of patient health information, whereas privacy is the freedom from unauthorized intrusion.

There are various types of mHealth apps; some collect health information from patients, whereas others simply provide general guidelines for maintaining a healthy lifestyle and information about certain diseases. If an mHealth app does not handle any patient health data, it typically does not trigger security and privacy concerns; therefore, the mHealth apps discussed in this study are the ones that handle patient health data.

Concern about health data security and privacy is one important reason people choose not to use mHealth apps for their own health care [3,9-11]. More specifically, users are not certain what type of data are collected and stored by mHealth apps, who can access the self-entered and sensor-collected data, and what purpose data are used for [10,12]. Security and privacy concerns about mHealth apps are greater when the apps are for issues associated with stigma, social isolation, or discrimination such as HIV/AIDS, sexual orientation, and mental disease [13-17]. All of these concerns are not surprising since millions of patients’ health records have been compromised because of hacking or other incidents in recent years in the United States [18]; many mHealth apps do not have the necessary security features to protect users’ health data [19-21]; at the same time, many smartphone users (including patients and health care providers) do not even use the most basic authentication features (such as a passcode) to prevent access to private data on their phones [22,23]. In addition, a recent study has indicated that in 2015 around 70% of the 600 most commonly used mHealth apps did not provide privacy policies; many current mHealth app developers do not provide privacy policies in their apps either [24,25].

One possible way to reduce users’ concerns about privacy in mHealth apps is to determine the specific concerns of mHealth app users, evaluate the association between these concerns and users’ characteristics (such as demographics, experience with technology, and health care needs), and identify specific mHealth app features that may enhance their trust in the apps so that they will start to use mHealth apps in their own health care and management. In other words, investigating the barriers to and facilitators of using mHealth apps may lead to finding a way to increase users’ adoption of mHealth apps. Before going into the details of this study, a brief review of previous studies on this topic is provided below.

### Previous Studies

A number of studies have been conducted to identify users’ attitudes toward and perceptions of mHealth apps using focus groups, questionnaires, and interviews [9,10,26-28]. Below is a summary of the findings in a few of these studies.

In 2015, Krebs and Duncan distributed a cross-sectional survey throughout the United States to determine the usage of mHealth apps among mobile phone owners and the reasons behind their choice about whether or not to use mHealth apps [9]. There were 1604 respondents in the study, and more than 40% of these mobile phone users reported that they had chosen not to download mHealth apps. One of the reasons given was security and privacy concerns. The ones who had chosen to download mHealth apps, on the other hand, seemed to trust in the security of the app. Individuals more likely to use health apps tended to be younger, have higher incomes, be more educated, be Latino/Hispanic, and have a body mass index in the obese range.

Atienza et al used a mixed-methods approach (survey and focus group studies) to determine consumer attitudes toward and perceptions of mHealth privacy and security [10]. The conclusion was that user attitudes regarding mHealth privacy and security were highly contextualized. They were related to the type, place, time, purpose, and person accessing the health information. They found that people in similar demographic groups may have quite different opinions on privacy.

Peng et al conducted focus groups and individual interviews with 44 smartphone owners to determine user perceptions of mHealth apps [26]. People in all demographic groups (age, gender, and income) revealed that they did not like to share health data in the app via social networking features. They had concerns about how the information might be exploited by a third party. They were willing to share selected information with a small number of people if necessary. Besides security and privacy concerns, one other major barrier the participants of the study mentioned was the cost of the app. Many people only used free mHealth apps.

Dennison et al conducted 4 focus group studies with students and staff at a university in the United Kingdom to assess the opportunities for and challenges to getting young adults to use smartphone apps in supporting health behavior change [27]. Study results indicated that young, healthy young adults have some interest in apps for behavior change. However, participants expressed concerns about the security of the data in the app. They were afraid that the data might get into the hands of third parties. They also felt it was intrusive when apps use a context-sensing approach to generate reminders or suggestions. They particularly did not like the app using the Global Positioning System to track their locations.

Prasad et al arranged 8 focus groups to identify privacy concerns related to mHealth apps [28]. The participants were young college students (aged between 19 and 30 years), elderly hospital outpatients (aged between 80 and 85 years), and residents of a retirement community (aged between 65 and 100 years). First, elderly participants were more comfortable sharing health information with their doctors than family members. The young participants were willing to share their medication information with their doctors but no other information such as their location and their social interactions with others. Second, some participants were afraid that some information collected by the mobile device might get compromised during transmission and storage. They wanted to have control over the disclosure of their data because they included private information. Young participants did not want the mobile device to collect their information without their consent.

One comprehensive review covered privacy in mobile technology for personal health care and discussed topics such as security regulations, technologies, threats, and possible solutions [29]. One review of privacy and security in mHealth apps briefly summarized the studies on security research in mHealth systems and provided general recommendations for creating secure mHealth apps [30]. Another study provided more detailed security recommendations for mHealth apps [31]. These recommendations are theoretical, with a major focus on the sensitivity of information itself. Although these recommendations for mHealth app developers are surely helpful in terms of making mHealth apps more secure, we also need to take end-user concerns into account and consider the usability of the mHealth app [32]. After all, no matter how secure the app is, if the end-users do not like it and do not use it, it will not contribute to the improvement of users’ health and well-being.

In other words, although there are several studies that have revealed the existence of security and privacy concerns from mobile app users, signiﬁcant diversity in attitudes regarding mHealth privacy/security exists for different demographic groups. Thus, one may need to specifically customize security features for different purposes and different users to address users’ individual concerns regarding mHealth privacy and security in apps.

### Objectives

In this study, we used a questionnaire and held interviews to assess the association between users’ demographic characteristics and their security and privacy concerns, and more importantly, the specific security features they desire to have in mHealth apps and the features or language that may encourage them to use mHealth apps.

The purpose of this questionnaire and interview study is to collect data and answer the following 4 questions:

1. What are mobile app users’ opinions or concerns about their personal data security and privacy?
2. What are mobile app users’ opinions and concerns about their data security and privacy in mHealth apps?
3. What are the security and privacy features they desire to see in mHealth apps?
4. What are the barriers to and facilitators of the use of mHealth apps?

## Methods

### Questionnaire and Interview Question Development

#### Step 1. Literature Search and Review

We used the literature collected in our previous research studies [33-38] and performed keyword searches “(security OR privacy)” AND “(questionnaire OR survey OR interview)” for published studies in PubMed, IEEE Xplore, ACMD Digital Library, and INSPEC. We also used the same keywords to perform searches in Google. From the obtained search results, we identified a few hundred statements relevant to information security and privacy.

#### Step 2. Creating a Draft of the Questionnaire

Each of the research team members went through these identified statements to determine their relevance and clarity in terms of the study purpose on a scale of 1 to 4, where 1 means no relevance or clarity, whereas 4 means high relevance or clarity. If 3 or 4 team members rated the relevance of a statement 1 or 2, it was removed from the questionnaire. If one of the team members rated the clarity of a statement 1 or 2, the wording of the statement was adjusted. The research team had multiple face-to-face meetings to discuss the rating and wording of statements. After this step, 24 statements remained in the questionnaire.

#### Step 3. Refining the Draft Questionnaire

The research team used the information from the literature and past experience to refine the draft questionnaire by adding, removing, and adjusting statements. Previous studies have indicated that users or patients are particularly interested in issues such as the locations at which their data are stored, who can access their data, the specific approaches used in handling their data, and the purpose for accessing their data [10]. Therefore, in this questionnaire, we specifically included questions related to these topics. There are many security and privacy features available in mobile apps, such as informed consent, privacy policy, access control in general, remote wipe, role-based access control, encryption, and multifactor authentication. Therefore, we created one statement for each of these topics as well. At the end of this step, there were 17 statements in the questionnaire, and they were arranged into 3 categories: opinion on personal data security and privacy, opinion on security and privacy in mHealth apps, and desired security and privacy features in mHealth apps.

We also wrote interview questions based on the information found in our literature review and experience gained from our work in the past. For instance, possible facilitators could be apps that are free and have a low data entry burden, a clear patient protection privacy policy, an intuitive user interface, and strong but easy-to-use security features, whereas possible barriers are the opposite of these desired features (eg, paid apps, heavy data entry burden, unclear privacy policy, hard-to-use user interface or security features).

#### Step 4. Pilot-Testing the Questionnaire

After we all agreed on the content validity of the statements and interview questions in this study, we then distributed the first version of the questionnaire and interview questions to 14 graduate students in an information security class. These graduate students reviewed this version and provided their comments on some statements and questions. We made changes on the statements and questions according to their suggestions. For instance, almost all the students indicated that before they took the information security class, they had had no idea what role-based access control was or did not know the details about encryption. Almost all of them also indicated that they did not like to use multifactor authentication even though they knew that feature would protect their highly sensitive information. A mobile app with multifactor authentication would simply discourage them from using the app. Therefore, statements corresponding to those security features (role-based access control, encryption, and multifactor authentication) were removed from the questionnaire because if respondents did not understand those features, the results obtained would not be reliable. The final questionnaire had 14 statements.

#### Step 5. Performing Questionnaire and Interview Studies and Psychometric Analysis

In this step, we recruited a group of study participants to conduct studies using these new questionnaire and interview questions. The obtained data were used to evaluate the reliability and validity of the new questionnaire and answer the research questions. The details of the study and the data analysis are presented in the following sections.

### Study Design

After the study protocol was approved by the institutional review board (IRB) office at the University of Pittsburgh, we recruited study participants with the following criteria: native English speaker, high school or higher education, aged between 18 and 65 years, capable of communicating with others orally and in writing, and has at least a few years of experience in using smart devices such as smartphone, tablet, or smart watch.

Study participants were recruited through flyers distributed in the Greater Pittsburgh area and through the Pitt + Me website at the University of Pittsburgh, which in January 2019 had more than 193,000 potential study participants registered at the site. Potential participants could indicate their interest in this study by sending a message to the research team or clicking on the link of the study on the Pitt + Me website. They were then screened according to the selection criteria. A list of these eligible subjects was stored in an Excel file. We then randomly selected study participants from this list to conduct the questionnaire and interview study.

Each study participant was given the opportunity to read and sign the IRB-approved consent form before the commencement of the study. At the beginning of the study, the investigators explained the purpose of the study, the procedure of the study, and the data to be collected in the study. Study participation was completely voluntary, and participants could stop participating in the study at any time.

During the study, the study participants were asked to provide answers to demographic questions, statements in the questionnaire, and the interview questions. When the study participants responded to the demographic questions and the questionnaire, the investigators did not provide any explanation on the terms used in the questionnaire. All of the answers to the questionnaire were collected with the Web-based Qualtrics system. When the study participants answered the interview questions, the investigators provided an explanation of some security terms if needed, such as encryptions, user authentication, multifactor authentication, access control, user auditing, and privacy policy. All the answers were noted, categorized, and entered into the Qualtrics system as well.

### Statistical Analysis

All statistical analyses were conducted using SPSS version 24 (IBM). The internal consistency of the questionnaire was evaluated using Cronbach alpha. For research or evaluation, a value of .7 to .8 in Cronbach alpha is considered reliable [39].

Descriptive statistics were calculated for all the items in the questionnaire and the interview questions. Statistical significance was determined by *P*<.05. The normality of the data was evaluated with the Shairo-Wilk test. Nonparametric Kruskal-Wallis H (KWH) test was used to determine the significance of differences among multiple categories.

## Results

### Demographics

In total, 117 participants were recruited in the Greater Pittsburgh area to undertake the survey and interview study. The demographic information is summarized in Table 1.

**Table 1:** Demographic characteristics of the study participants (N=117).

Demographics		Statistics
Age (years), mean (SD)		31.49 (12.354)
**Age (years), n (%)**		
	18-28	64 (54.7)
	29-50	39 (33.3)
	51-65	14 (12.0)
**Gender, n (%)**		
	Male	53 (45.3)
	Female	64 (54.7)
**Race, n (%)**		
	African American	16 (13.7)
	White	76 (65.0)
	Asian	25 (21.4)
**Education, n (%)**		
	Below bachelor’s	39 (33.3)
	Bachelor’s	44 (37.6)
	Graduate	34 (29.1)
**Marital status, n (%)**		
	Single	80 (68.4)
	Married	34 (29.1)
	Divorced or separated	3 (2.6)
**Occupation, n (%)**		
	Student	37 (31.6)
	Health care provider	9 (7.7)
	Customer service	19 (16.2)
	Administrative personnel	14 (12.0)
	Researcher	14 (12.0)
	Other	24 (20.5)
**Self-assessed health status, n (%)**		
	Excellent	30 (25.6)
	Very good	49 (41.9)
	Good	29 (24.8)
	Fair	9 (7.7)
Years of using mobile devices, mean (SD)		6.21 (2.585)
**Years of using mobile devices, n (%)**		
	<3	11 (9.4)
	3-5	36 (30.8)
	>5	70 (59.8)
**Used mHealth apps, n (%)**		
	Yes	79 (67.5)
	No	38 (32.5)
**Household income, n (%)**		
	<US $10,000	20 (17.1)
	US $10,000-US $75,000	62 (53.0)
	>US $75,000	22 (18.8)
	Decline to answer	13 (11.1)

### Responses to the Statements on the Questionnaire

There were 14 statements on this questionnaire, and the study participants were required to select an answer from 1 to 7, corresponding to strongly agree (1), agree (2), somewhat agree (3), neither agree nor disagree (4), somewhat disagree (5), disagree (6), and strongly disagree (7).

The Cronbach alpha of this 14-item questionnaire was .730, which is good for research and exploratory studies. The overall mean for all 14 items was 2.55 (SD 0.658), reflecting that most study participants agreed with these statements to a certain degree. These 14 items were categorized into 3 groups: opinions on personal data, opinions on mHealth apps, and security features that users desire in mHealth apps.

The first category, opinion on personal data security and privacy, had 5 statements: S1, S2, S3, S4, and S5. The Cronbach alpha of this category was .737. The overall mean of the first category was 2.72 (SD 1.06), indicating that the study participants somewhat agreed with the 5 statements about personal information. They had some level of concern about the privacy of their personal data and wanted to have some specific protections on their personal data.

Similarly, the second category, opinion on security and privacy in mHealth apps, had 5 statements as well: S6, S7, S8, S9, and S10. The Cronbach alpha of this category was .785. The overall mean of the second category was 2.78 (SD 1.05), which indicates that the study participants generally accepted using mHealth apps for health care purposes, and most of them also believed that there was some level of privacy protection currently available in mHealth apps.

The third category of statements was about several security and privacy features in mHealth apps, such as informed consent (S11), access control (S12), privacy policy (S13), and remote wipe (S14). These statements fall into the same category in general; however, they are not in the same construct since each of them reflects a specific aspect of security. Therefore, it was not surprising to see that the Cronbach alpha of this category was .346. The overall mean of this group was 2.06 (SD 0.547), which reflects that the study participants desired to have those features in mHealth apps.

Table 2 provides a descriptive summary of the answers to the statements in this questionnaire. A mean of less than 4 means that these study participants agreed with the statement; the smaller the value, the stronger the agreement. A mean of greater than 4 means that the group disagreed with the statement; the bigger the value, the stronger the disagreement. The numbers in Table 2 indicate that these 117 study participants generally agreed with almost all of the statements, some showing stronger agreement, and some showing weaker. The only exception is the reported opinions on the privacy policy. It seems that many study participants did not believe that the content of the privacy policy of a mobile app could influence their decision with respect to app selection. This may be related to the readability of mHealth app privacy policies [38,40,41]. At the same time, almost all of the study participants desired to have the other 3 security features (informed consent, access control, and remote wipe) included in mHealth apps.

### Relationship Between Demographic Characteristics and Answers to the Statements

The Shairo-Wilk test on the participants’ answers to the 14 statements indicated that the data were not normally distributed (*P*<.05 in all cases). Therefore, the nonparametric KWH test was used to determine the relationship between study participants’ demographic characteristics and their responses to the statements in the questionnaire. The differences were not statistically significant for people with different education levels (below bachelor’s, bachelor’s, and graduate), health status (excellent, very good, good, and fair), occupations, years of using mobile devices (<3, 3-5, >5), or employment status (employed vs unemployed).

#### Marital Status

In general, married participants had stronger concerns about information security and privacy and desired to have more stringent security protection. Correspondingly, they also were not very comfortable with their health provider using mHealth apps to manage their health data. However, in most cases, these differences were not statistically significant. A KWH test showed that there was a statistically significant difference in statement S2 between those of different marital status, KWH (1)=4.8, *P*=.03, with a mean rank score of 61.84 for single participants and 47.28 for married participants. A lower value corresponds to stronger agreement with the statement.

#### Sex

In most statements, the values from males and females were different, but these differences were not statistically significant (*P*>.05). The difference was statistically significant on S11 (I should have the right to consent to any sharing of my protected health information collected via mHealth apps). There, the mean rank was 67.93 for male and 51.60 for female, *P*=.001, KWH (1)=11.3, indicating that females have a stronger desire to have the right to consent to their health data collection.

**Table 2:** A summary of responses to the statements in the questionnaire. Here, agree corresponds to 1 to 3, neutral corresponds to 4, and disagree corresponds to 5 to 7 (N=117; overall Cronbach alpha=.730).

Statements		Agree (1-3), n (%)	Neutral (4), n (%)	Disagree (5-7), n (%)	Mean (SD)
**Opinion on personal data (Cronbach alpha=.737)**					
	S1. In general, I am concerned about the privacy and security of my personal information in everyday life	97 (82.9)	7 (6.0)	13 (11.1)	2.46 (1.424)
	S2. I am concerned about the privacy and security of my personal information l when using an mHealth app	82 (70.1)	12 (10.3)	23 (19.6)	2.91 (1.710)
	S3. I am concerned about submitting personal information on an mHealth app because of what others might do with it	67 (57.2)	16 (13.7)	34 (29.1)	3.38 (1.731)
	S4. I do not want to store my personal identifiers (such as name, SSN, phone number, email address) in the mHealth apps except for one unique ID number which is only recognizable by authorized personnel	87 (74.4)	19 (16.2)	11 (9.4)	2.65 (1.422)
	S5. I would like my personal health data to be transferred to a centralized database via a highly secure process	101 (86.3)	11 (9.4)	5 (4.3)	2.19 (1.245)
**Opinion on mHealth apps (Cronbach alpha=.785)**					
	S6. Overall, I am satisfied with the privacy and security of the mHealth apps I am currently using	80 (68.4)	28 (23.9)	9 (7.7)	2.82 (1.317)
	S7. Health care providers have the necessary security and privacy measures in place. These measures provide a reasonable level of protection for information collected from mHealth apps	79 (67.5)	20 (17.1)	18 (15.4)	3.02 (1.333)
	S8. I would use mHealth apps for my health care needs	95 (81.2)	10 (8.5)	12 (10.3)	2.50 (1.369)
	S9. I want my health care providers to use mHealth apps to store and manage my health information	71 (60.7)	27 (23.1)	19 (16.2)	3.05 (1.479)
	S10. I would feel comfortable if my health information was shared among my doctors and therapists for my health care purpose	94 (80.3)	7 (6.0)	16 (13.7)	2.52 (1.643)
**Desired features in mHealth apps (Cronbach alpha=.346)**					
	S11. I should have the right to consent to any sharing of my protected health information collected via mHealth apps	114 (97.4)	1 (0.9)	2 (1.7)	1.39 (0.861)
	S12. I would like to know how my health care providers make sure that only the correct personnel have access to the mHealth system I am using	111 (94.9)	6 (5.1)	0 (0)	1.56 (0.814)
	S13. I read the privacy policies of mHealth apps. The content of the policies influences my decision of whether to use the app	51 (43.6)	15 (12.8)	51 (43.6)	3.89 (1.902)
	S14. I would like to be able to remotely remove all my health data on my mobile device if it is lost or stolen	114 (97.4)	2 (1.7)	1 (0.9)	1.39 (0.719)

#### Race

As shown in the demographic information, three races were represented in this study: African American, white American, and Asian American. In most (13/14, 93%) of these statements, the responses among these three races do not have a statistically significant difference. There is a statistically significant difference between race groups for one statement (S1) as determined by KWH test, *P*=.01, χ^2^_2_=8.5. The mean rank was 49.69 for African Americans, 65.42 for white Americans, and 45.55 for Asian Americans. In other words, Asian Americans have a significantly stronger privacy concern than white Americans in general.

#### Household Income

The study participants were arranged in 4 groups according to their household income: less than US $10,000, between US $10,000 and US $75,000, greater than US $75,000, and decline to answer. In most statements (12/14, 86%), answers from the 4 groups were similar, but answers to 2 statements (S2 and S3) have statistically significant differences. For S2, χ^2^_3_=8.9, *P*=.03 and for S3, χ^2^_3_=9.4, *P*=.02. Study participants with less than US $10,000 annual income had the weakest concerns about security and privacy. They were satisfied with the security and privacy protection provided by current mHealth apps. Therefore, they were willing to use mHealth apps themselves and also wanted their health care providers to use mHealth apps to manage their data and share their data with other doctors or therapists for health care purposes. They did not make selections on mHealth apps according to privacy policy, but they still in general agreed that data should be transmitted securely. Study participants with greater than US $75,000 annual income had the strongest concerns about and desire for security and privacy. They also expected the most stringent security measures to protect their privacy, such as the right of consent, access control, limited data stored, highly secure data transmission, and ability to remotely remove personal data if their mobile device is lost or stolen.

#### Age Groups

The study participants were arranged into 3 age groups: 18 to 28 years, 29 to 50 years, and 50 to 65 years. The general trend of the answers from these age groups was obvious. Participants in the 51 to 65 years age group had the strongest concern about privacy in mHealth apps and were willing to use mHealth apps for health care purposes but expected to have strong security protection in mHealth apps. Participants in the 18 to 28 years age group had the weakest concern about privacy, were somewhat satisfied with current security protection, and believed that health care providers took the necessary security and privacy measures to provide that protection. They were willing to use mHealth apps in their health care but not as strongly willing as those in the other 2 age groups. Therefore, their desire to strengthen security and privacy measures was relatively weaker compared with the other 2 age groups, but they still desired to have those measures strengthened because they did have concerns about privacy. Participants in the 29 to 50 years age group were somewhere in the middle. They had privacy concerns, did not believe the current practice was sufficient, did not have a strong desire to use mHealth apps in health care; therefore, in their response to the statements, they did not show strong desire to strengthen the security measures in mHealth apps. The differences among these age groups in multiple statements (S3, S5, S6, S8, S10, S11, S12, and S14), however, were not statistically significant.

The KWH test showed that there were statistically significant differences in answers to 6 statements (S1, S2, S4, S7, S9, and S13) among the different age groups (AG1: 18-28, AG2: 29-50, AG3: 51-65). Table 3 shows the test statistics and the mean rank for AG1 and AG3.

#### Experience Using Mobile Health Apps

In most cases, the means from participants who had used mHealth apps before were smaller, which indicated that these participants had a stronger concern about security and privacy in mHealth apps, but they still wanted to use mHealth apps for their health care needs and desired to have stringent security. Most of these differences were not statistically significant. These 2 groups (participants who had used or had not used mHealth apps before) had a statistically significant difference for one statement: S8 (I would use mHealth apps for my health care needs). The mean rank from the participants who had used mHealth apps before was 54.25, whereas that from the participants who had not used mHealth apps before was 68.87. Here KWH (1)=5.1, *P*=.02. In other words, participants who had used mHealth apps before still wanted to use mHealth apps for their health care needs, whereas participants who had not used mHealth apps before were still hesitant to commit to such a decision.

### Answers to Interview Questions

The first interview question was about password change frequency. Nine (9/117, 7.7%) participants responded they would like to change their passwords every month; 44 (37.6%, 44/117) participants indicated a willingness to change their passwords every 3 months; 33 (28.2%, 33/117) participants accepted changing their passwords every 6 months; 23 (19.7%, 23/117) participants claimed to be willing to change their passwords once a year; 2 participants (1.7%, 2/117) would rather change passwords once every 2 years; and 5 participants (4.3%, 5/117) indicated that they would never change their passwords. One participant stated that he would rather not change it regularly but would like to make the change when he believed necessary.

**Table 3:** Test statistics in the Kruskal-Wallis test on 6 statements among 3 different age groups.

Statement	Chi-square (*df*)	*P* value	Age groups (mean rank)	
			Age group 1	Age group 3
S1	11.2 (2)	.004	67.36	38.29
S2	16.1 (2)	<.001	69.03	33.18
S4	6.7 (2)	.04	61.04	37.68
S7	7.1 (2)	.03	63.24	37.43
S9	7.5 (2)	.02	64.8	38.39
S13	8.9 (2)	.01	67.37	47.11

**Table 4:** Barriers to and facilitators of the use of mobile health (mHealth) apps identified in the semistructured interview study (N=117).

Questions and answers		n (%)	
**Q1. What barriers would prevent the adoption and integration of a mHealth App into your health monitoring and management?**			
	Price of mobile apps. I only use free mobile health apps.		78 (66.7)
	The app sends my data to a remote server without my permission.		73 (62.4)
	The app asks me to provide my personal information even when I just want to determine whether the app is good for me.		71 (60.7)
	The app does not encrypt my personal data.		69 (59.0)
	The app runs slowly even though my mobile device is a recent model.		67 (57.3)
	The app does not have clear privacy statements about how it handles my personal data.		59 (50.4)
	The app stores my personal data on my mobile device and makes the data easily accessible to anyone who can access my mobile device.		58 (49.6)
	The app sends me several alerts each day.		56 (47.9)
	Name of mobile app. For instance, I do not use an app where the name implies that I have a certain disease.		48 (41.0)
	Price of mobile app. I only use mobile health apps costing less than $5.		26 (22.2)
	The app asks me to set up an account with user name and password.		14 (12.0)
	Other (eg, The app has two-factor authentication. The app requires social network login.)		3 (2.6)
**Q2. What security measures would give you confidence that an mHealth app would protect the confidentiality of patient data?**			
	Explicit encryption on data stored on my mobile device and the data transmitted to a remote server.		96 (82.1)
	User authentication.		96 (82.1)
	Remote removal of my personal data on a lost mobile device.		96 (82.1)
	Access control.		92 (78.6)
	Easy-to-understand privacy policy which clearly indicates that my personal data are well protected.		91 (77.8)
	Data transmission via a secure channel.		74 (63.2)
	Easily adjustable security settings for different types of data.		74 (63.2)
	All health care providers’ data access activities are logged and can be audited.		70 (59.8)
	One unique account for each patient and each health care provider.		67 (57.3)
	Regular password update.		51 (43.6)
**Q3. What specific privacy policies of an mHealth app would encourage you to use the app for your own health care purpose?**			
	Your data will NOT be shared with any unauthorized personnel.		98 (83.8)
	Your data will be collected only if you give permission to the app.		94 (80.3)
	Your data will be removed from the server if you request it.		93 (79.5)
	You have the right to terminate the permission for data collection at any time.		90 (76.9)
	Your data will be collected only for health care and/or research purposes.		78 (66.7)

The second interview question was about whom they were willing to share their medical information with. Participants could mention people from multiple categories. One hundred and nine (109/117, 93.2%) participants indicated that they would share their medical information with their health care providers; 81 (69.2%) participants with their family members; 38 (32.5%, 38/117) participants with friends; 12 (10.3%, 12/117) participants at password-protected personal websites, patient support groups, or password-protected online patient forums for patients with similar conditions; and 4 (3.4%, 4/117) participants claimed that they were not willing to share their medical information with anyone else.

The last 3 interview questions were about barriers to and facilitators of the use of mHealth apps. The answers from the study participants are summarized in Table 4.

Cost was a significant barrier among respondents, with a large proportion (78/117, 66.7%) indicating that they would not pay anything for a health app. Other barriers mentioned by more than half of the study participants were lack of encryption and informed consent, poor app performance, and request for personal information during app testing stage. Some study participants also mentioned issues such as unclear policy statements, too many alerts, name of mobile app (for instance, some app names include the name of a disease), and inconvenient user authentication.

The study participants also listed a number of facilitators in terms of mobile app security and privacy features, such as encryption, user authentication, user auditing, remote wipe, a clear user protecting privacy policy, and flexible security settings. Specific to the language of a privacy policy, the study participants indicated that they would like to see that they need to provide permission before the data are collected, to know the specific purpose for collecting the data, and to have the ability to stop the data from being collected.

## Discussion

### Principal Findings

In this study, we first confirmed that mobile app users had security and privacy concerns when they used mHealth apps in their daily life and identified the level of these concerns in people with different demographic characteristics such as sex, age, gender, education, income, and experience using mobile devices; some differences were statistically significant, whereas others were not. These results are consistent with the results reported in a number of previous studies [9,10,26-28] and the theory of privacy as contextual integrity [42]. Specific to mHealth app development, it means mHealth app developers may need to make certain adjustments to mHealth apps’ security features for different user groups in terms of marital status, sex, age, and income.

More importantly, we identified the security features desired in mHealth apps using a questionnaire and interview questions, the encouraging language in privacy policies, and the specific barriers in the mHealth app adoption. The findings can be used to guide the design and development of new mHealth apps. In previous studies [9,10,26-28], the major focus was on eliciting and reporting security and privacy concerns, although the recommendations for mHealth app development were typically brief, and even if they were available, they were not from the research studies themselves but general principles of information security. The frequently mentioned security approaches were requiring user authentication (eg, password), information hiding, and informed consent [14,16]. For previous studies that did provide highly detailed security feature recommendations for mHealth app development [31], those recommendations were theoretical in terms of information security itself and did not take the user’s characteristics into consideration. The results of this study offer a clearer picture in terms of security and privacy features desired by users with various characteristics in mHealth apps.

One may argue that today’s smartphones already have a number of security features implemented; these include data encryption, device password lock, remote data wipe, remote device locator, and antimalware apps. Moreover, a good use of these security features can provide strong protection to users’ privacy and their sensitive data such as health records; however, a recent questionnaire study involving 458 smartphone users clearly indicated that most smartphone users do not use these security features [23], and therefore, the task of data protection still falls to mHealth app developers.

According to the results of the study, the vast majority (111/117, 94.9%) of study participants desired to know how health care providers apply access control to their health data. In other words, they wanted to make sure that only authorized personnel could access their health data. The remaining 6 participants (5.1%, 6/117) did not indicate a preference on this issue. This is a topic related to patient education via the mHealth app. In other words, how can an mHealth app convince users that only authorized personnel can access their health data? To address this concern or answer this question, mHealth app developers need to demonstrate to users that corresponding security and privacy features are included in the mHealth apps.

In this study, most of the study participants had good health status even though we did not use health status as one of the selection criteria during screening. We understand that the findings could be significantly different if those with serious health problems and a strong desire to take advantage of the convenience offered by mHealth apps had been part of the population. For instance, patients who have experienced heart failure or who have had a kidney transplant have been shown to welcome mobile app–based home monitoring and reminder systems [7,43-45]. This is very common in the field of security and privacy. People perform their own risk and benefit assessment when they face a choice. The benefits provided by mHealth apps can be free cost, convenience, real-time health services, saving time, and other monetary incentives [46,47]. If users believe that the benefits outweigh the security and privacy risks, they may choose to sacrifice privacy and enjoy the benefits offered by the service, even though they still have concerns.

### Limitations

The study was performed at the University of Pittsburgh, and the study participants were recruited from the Greater Pittsburgh area. Roughly one-third of the study participants were undergraduate and graduate students, and they were from many different states. Therefore, the opinions reported in this study can also reflect the opinion of people in other states of the country, or at least people in that specific age group.

The sample size of this study was not very big, but it was sufficient for the purpose of this study. In most demographic categories, the number of participants was sufficient for the analysis. We believe the current sample size is big enough for us to obtain reliable results since these participants were randomly selected from a few hundred potential participants who explicitly expressed their interest in this study. On the other hand, a larger sample size would make the results more convincing and increase the generalizability of the results.

Most of the study participants were young and healthy. Therefore, we did not see a significant difference in opinion with respect to privacy among health status groups. As we did not have very sick people included in this study, the results cannot be generalized to that population.

This study was performed in the United States, and some results may not be applicable in other countries, especially in countries with significantly different regulations and culture. For instance, in many Asian countries, a family member’s health information is openly shared with family members. Employees are also typically required to have an annual physical exam, and the results are reported to employers. Therefore, people in this situation typically do not have a strong privacy concern since there is no corresponding protection anyway.

### Future Research

In the future, we will create an mHealth app with those highly desired security and privacy features identified in this study, and we will determine whether that changes app users’ trust in the app. We will also perform analysis on the usage data and their security settings to determine whether they have utilized those security features, whether and how the actual app usage has changed, and which specific security features they commonly choose to disable.

It is also necessary to enhance the security education to mobile app users so that they are well aware of the many readily available security features on their smartphones and can take advantage of these features to protect their data and privacy [38].

## Abbreviations

AG: age group
IRB: institutional review board
KWH: Kruskal-Wallis H
mHealth: mobile health
NIDILRR: National Institute on Disability, Independent Living, and Rehabilitation Research
NSF: National Science Foundation
